# The heterogeneous clinical and pathological landscapes of metastatic *Braf*-mutated colorectal cancer

**DOI:** 10.1186/s12935-020-1117-2

**Published:** 2020-01-29

**Authors:** Giuseppe Nicolò Fanelli, Carlo Alberto Dal Pozzo, Ilaria Depetris, Marta Schirripa, Stefano Brignola, Paola Biason, Mariangela Balistreri, Luca Dal Santo, Sara Lonardi, Giada Munari, Fotios Loupakis, Matteo Fassan

**Affiliations:** 1 Surgical Pathology Unit, Department of Medicine (DIMED), University of Padua, via Gabelli 61, 35121 Padua, Italy; 2 Department of Oncology, Veneto Institute of Oncology IOV–IRCCS, Padua, Italy

**Keywords:** *BRAF* mutation, Colorectal cancer, Personalized medicine, Sequencing

## Abstract

Colorectal cancer (CRC) is a complex and molecularly heterogeneous disease representing one of the most frequent causes of cancer-related death worldwide. About 8–15% of CRCs harbor a mutation in *BRAF* gene, a proto-oncogene involved in cell proliferation, differentiation and survival through the MAPK signaling cascade. The acquisition of *BRAF* mutation is an early event in the “serrated” CRC carcinogenetic pathway and is associated with specific and aggressive clinico-pathological and molecular features. Despite that the presence of *BRAF* mutation is a well-recognized negative prognostic biomarker in metastatic CRC (mCRC), a great heterogeneity in survival outcome characterizes these patients, due to the complex, and still not completely fully elucidated, interactions between the clinical, genetic and epigenetic landscape of *BRAF* mutations. Because of the great aggressiveness of *BRAF*-mutated mCRCs, only 60% of patients can receive a second-line chemotherapy; so intensive combined and tailored first-line approach could be a potentially effective strategy, but to minimize the selective pressure of resistant clones and to reduce side effects, a better stratification of patients bearing *BRAF* mutations is needed. 
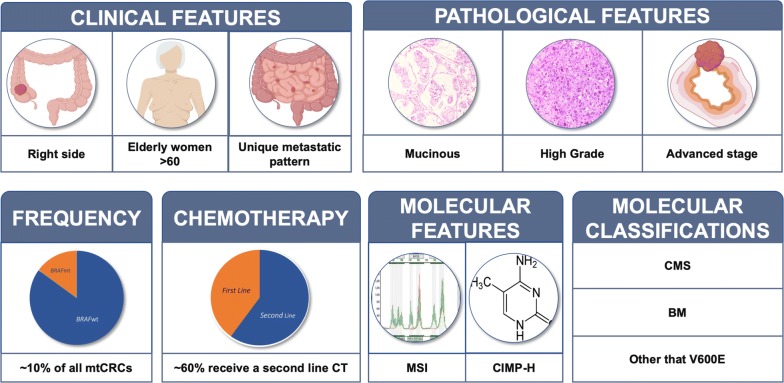

## Background

Colorectal cancer (CRC) is the fourth most common malignancy and the second most frequent cause of cancer-related death worldwide [1]. CRC is a complex and molecularly heterogeneous disease, characterized by different genomic landscapes [2, 3]. CRC phenotypic and molecular comprehensive characterization represents a key step, with diagnostic, prognostic and predictive value both in localized and in metastatic settings (mCRC) [4].

Among the most frequent mutations with prognostic and predictive value, missense point mutations in *BRAF* gene occur in about 8–15% of mCRC, being mutually exclusive with *RAS* genes mutations [5].

*BRAF* (v-raf murine sarcoma viral oncogene homolog B) encodes for a protein kinase acting through the MAP (mitogen-activated protein) kinase cascade, playing an important role in cell proliferation, differentiation and survival [6]. Given its pivotal location in many neoplastic-related dysregulated pathways, it easily explains its oncogenic role in many human malignancies, including melanoma, ovarian carcinoma, papillary thyroid carcinoma and CRC [7, 8]. Of note, the oncogenic contribution of mutated *BRAF* gene varies between cancer types, justifying significant differences in clinico-pathological features, prognostic impact and therapy response among various malignancies [9–13].

**Fig. 1:**
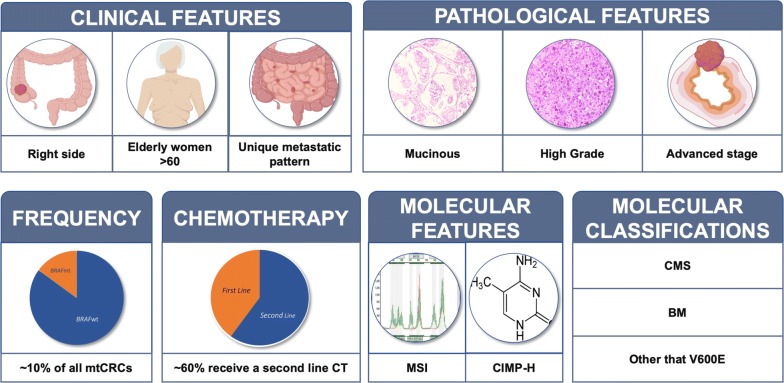
Clinico-pathological features associated to *BRAF*-mutated colorectal cancers

*BRAF*-mutated (*BRAF*mt) CRCs have specific clinico-pathological and molecular features [14–18], identifying a distinct subset with aggressive phenotype and poor outcome (Fig. 1). Noteworthy, *BRAF*mt tumors are more frequent in elderly persons with scant performance status. By the pathological point of view, *BRAF*mt CRCs are characterized by more aggressive pathological features like high grade and peritoneal dissemination and they present in an advanced stage at the time of the initial diagnosis. Furthermore, *BRAF*mt mCRC patients usually develop an early resistance to standard and targeted-therapy, and only about an half of these patients can receive a second line chemotherapy, suggesting that more aggressive and individualized combined therapies may be effective in selected patients cohorts [7, 15, 19–24]. Nevertheless, *BRAF* treatment-predictive value still remains a matter of debate.

A strong association between *BRAF* mutation and microsatellite instability (MSI) has been shown (46–75%) [25–28]. This finding is consistent with the evidence that most *BRAF*mt CRCs develop via the “serrated pathway”, often followed by a “CpG-island methylator phenotype” (CIMP) involved in promoter methylation and silencing of key onco-suppressor genes [29]. Moreover, MSI CRCs share some clinico-pathological features with the *BRAF*mt ones [27]. By contrast, a small amount of CRCs harbor a *BRAF* mutation but remain microsatellite-stable (MSS), resulting in a worse prognosis than *BRAF*mt/MSI cancers [30, 31]; however, molecular characterization and predictive value of this particular subgroup has not yet been fully clarified. Because of the above mentioned prognostic and predictive implications, the molecular profiling of *RAS* (*KRAS* and *NRAS*) and *BRAF* genes and the assessment of Mismatch Repair (MMR)/MSI status has now been added into the main CRC diagnostic and therapeutic algorithms [32].

Of note, many studies have demonstrated that the *BRAF* negative prognostic impact is often independent of the other considered clinico-pathological features [33]. This could be related to several factors: different *BRAF* mutations have different prognostic value [34]; CRC intratumor heterogeneity and the complex interactions with other molecular alterations can influence the therapeutic response; *BRAF* mutation in CRC is difficult to target and several resistance mechanisms have been discovered, but some of them still remain unknown; tumor stage can influence the prognostic value of *BRAF* mutations.

Despite remarkable advances in CRC molecular classification have been made, the abovementioned aspects underline a still unsatisfied need: a reliable prognostic and predictive stratification for CRC patients that harbor a *BRAF* mutation. All of these aspects will be fully analyzed in the present review, in order to provide a comprehensive overview on current clinico-pathological, prognostic and predictive implications of *BRAF* mutation in CRC.

### *BRAF* gene and its pathway

*BRAF* is a proto-oncogene that encodes for a cytoplasmic serine/threonine kinase (STK), an essential component of the RAS/RAF/MEK/ERK signaling cascade [35]. Physiologically, extracellular soluble factors like EGF (Epidermal Growth Factor) bind and activate EGFR (Epidermal Growth Factor Receptor), a Receptor tyrosine kinases (RTK). Through the recruitment of two adaptor proteins (SOS and GRB2) EGFR activation allows KRAS to release GDP and to bind GTP. After some conformational changes, KRAS recruits and binds the cytosolic BRAF, which forms an active homo- or hetero-dimer with other component of the RAF family proteins. This homo/hetero-dimer phosphorylates and activates MEK kinases (MEK1 and MEK2) and, finally ERK, which translocates in the nucleus, stimulating transcription factors involved in proliferation, differentiation, cell motility, apoptosis (regulating BCL-2) and survival (through the HIPPO pathway) [5, 35].

### *BRAF* mutations and CRC clinico-pathological features

Different *BRAF* point mutations can affect the encoded protein function in many ways; most of them cluster to the conserved P loop and DFG motif of the kinase, destabilizing the inactive protein structure and thereby promoting an active conformation [34, 36].

The most common *BRAF* mutation (90%), in CRC as well in others malignancies, is a CTG → CAG transversion at residue 1799 (T1799A), leading to an amino acidic substitution from valine to glutamic acid at codon 600 (p.V600E) in the exon 15 (^V600E^*BRAF)*. This results in a constitutive-active kinase, 500-fold more active than BRAFwt [36]. Among all malignancies, V600E mutation occurs in 8–10% of all cancers (melanoma 66%, papillary thyroid cancer 53%, serous ovarian cancer 30% and 8–15% of sporadic CRCs) and it is often associated with poor prognosis [37, 38].

^V600E^*BRAF* mutation prevalence in CRCs is different among ethnic group (Asian population shows a lower frequency compared with Caucasian population) [39] and tumor-stage (^V600E^*BRAF* mutation frequency is significantly higher in stage II/III than in stage IV) [40]. However, its prognostic value in early disease is still controversial and should be further investigated [40, 41].

mCRCs harboring ^V600E^*BRAF* mutations have distinct clinico-pathological features compared to *BRAF*wt ones, outlining a unique (and often easily recognizable) subgroup [14, 42]: clinically, *BRAF*mt mCRCs arise in older patient (> 60 years old); this is in line with the evidence that *BRAF* mutations are an acquired genetic event, occurring mainly in sporadic CRCs and are unlikely to be common in younger patients [42]. *BRAF*mt mCRCs are prevalent in the female gender, regardless of the MSI status [42]; proximal colon is the preferential location, suggesting that the genomic alterations in proximal and distal colonic mucosa produce different CRC phenotypes [42]. Moreover, *BRAF*mt mCRCs present a unique metastatic pattern, showing high rates of peritoneal metastases, distant lymph node metastases [15, 27, 43–45] and low rates of lung metastases [15, 27]. Instead, no significant differences have been seen in liver or brain metastases rates [27]. Finally, ^V600E^*BRAF* mCRC patients usually have worse performance status (PS 1-2 using Eastern Cooperative Oncology Group scale) at first diagnosis [44, 45].

^V600E^*BRAF* CRCs have histological hallmarks that are widely reported in literature, such as mucinous features, serrated architecture, poor differentiation and high stage at diagnosis [14, 16, 23, 25, 42–46]. Other less peculiar features are a higher frequency of tumor budding and signet ring cells, infiltrative pattern of invasion with an increased risk of lympho-vascular but not perineural invasion, different grade of Tumor Infiltrating lymphocytes (TILs) and of peritumoral lymphoid reactions (Crohn-like) [16].

In addition, Chen et al. [14] explored the association between *BRAF* mutations and behavioral risk factors for CRC development. Despite these analyses were affected by the lack of data, is apparently excluded an association between ^V600E^*BRAF* mutation and smoking or dietary habits, while alcohol intake seemed to act as a protection factor against the same mutation.

### *BRAF* mutations and CRC carcinogenesis

Several molecular, morphologic and clinico-pathologic features have been studied to classify CRC and its carcinogenetic pathways, in order to better stratify patients and guide their therapeutic management. Three genomic pathways essentially drive CRC carcinogenesis: (i) the chromosomal instability (CIN) or aneuploidy pathway, which causes numerous changes in chromosomal copy number and structure; (ii) the microsatellite instability (MSI pathway, with loss of MMR function; and (iii) the “CpG island methylator phenotype” (CIMP) characterized by epigenetic instability due to methylation and silencing of critical tumor suppressor genes [2, 15, 47–49], a great overlap exists between the last two pathways.

Phenotypically, CRC pathogenesis has been described as a multistep process and two alternative pathways lead to sporadic invasive cancer: (i) the “conventional pathway” (tubular and tubulovillous adenoma → carcinoma) and (ii) the “serrated pathway” (microvesicular and goblet cell hyperplastic polyp → sessile serrated and traditional serrated adenoma → carcinoma). The three abovementioned genomic instability pathways are differentially involved in these two different progression models [15, 50].

The “conventional” pathway (70–80% of sporadic CRCs), is linked to CIN and is characterized by an early *APC* truncating mutation and subsequent alterations in *SMAD4* and *TP53* genes for the tubular adenoma (CRC *BRAF*wt/*KRAS*wt/MSS) and also in *KRAS* gene for tubulovillous adenoma (CRC *BRAF*wt/*KRAS*mt/MSS) [5, 51].

The “serrated” pathway (20–30% of sporadic CRCs) is related to MSI and CIMP [5, 51] and could be split in two routes according to the precursor lesions and mutational status of *BRAF* and *KRAS* genes. The former gene mutations occur frequently in microvesicular hyperplastic polyp (MVHP) and in sessile serrated lesion (SSL), are linked to “CIMP-High” phenotype and could be associated with either MSS or MSI status (CRC: *BRAF*mt/CIMP-H/MSI or *BRAF*mt/CIMP-H/MSS); whereas the latter have a higher incidence in goblet cell hyperplastic polyp (GCHP) and in traditional serrated adenoma (TSA), is linked to “CIMP-Low” phenotype and have a MSS status (CRC *KRAS*mt/CIMP-L/MSS) [52].

Hyperplastic polyps (HPs) represent more than 75% of the serrated polyps [35], they can be endoscopically recognized as flat or sessile polyps, pale in color, generally < 5 mm, and usually located in the distal colon (85–90%). However, only two HPs subgroups (MVHPs and GCHPs) might rarely acquire transforming and progression capacity [53, 54].

MVHPs are typically located in the proximal colon and are histologically composed of funnel-shaped crypts with serrations limited to upper two-thirds, the proliferation zone is located uniformly in the basal portion of crypts and cytologically have small basally located nuclei without dysplasia. Whether SSL arise de novo or from MVHP still remains debated and the molecular driver of this transition is unclear. However, MVHP and SSL have overlapping molecular alterations, both harbor an activating *BRAF* mutation, but only 10% of MVHPs have CIMP-High phenotype versus 40–50% of SSLs [51].

GCHPs could be located in proximal or distal colon and are histologically characterized by elongated crypts that resemble enlarged normal crypts with little/no serrations composed by cells with small and basally located nuclei without dysplastic hallmarks and the proliferation zone located at the cryptal basis. A GCHP can sometimes be found on the ‘shoulder’ area of TSA, and *KRAS* mutation is present in 43–54% of GHCPs suggesting that *KRAS* mutated TSAs may arise from this subgroup of HPs [51].

SSLs (20–25% of the serrated polyps) [52] are the most relevant serrated polyps, since they are the precursors of the largest proportion of CRCs developed through the serrated pathway. Macroscopically, they appear as flat or slightly elevated areas of mucosa, often located in the proximal colon and generally sized > 5 mm with a yellow mucous cap that often make the endoscopic diagnosis easier [54, 55]. The histologic hallmarks of SSL are the serrations extended into the crypt base that are horizontally enlarged along the *muscularis mucosae*, creating characteristic asymmetric structures (T- or inverted L-shaped) [51]. Of note, only 2–5% of SSLs (< 0.5% of serrated polyps) progress towards invasive CRCs and the hallmark of this progression potential is the dysplasia [56–58] characterized by architectural changes (crowded and elongated crypts with increased branching complexity, cribriforming, and villous architecture) and cytological atypia (from subtle hypermucinous changes to overt dysplastic changes) [59]. The molecular landscape of SSLs is characterized by wild type *KRAS*, mutated *BRAF*, CIMP-H phenotype (acquired in early phase) with methylated *MLH1* which result in MSI or methylated *MGMT* with MSS status, WNT pathway activation and in a minority of cases p16 silencing and *TP53* mutation (acquired in late phase with dysplasia) [60].

Only 5% of serrated polyps are TSAs. They range from 9 to 14 mm in maximum dimension, endoscopically have a “pinecone-like” appearance and a proximal or distal location; the three histological hallmarks are: the slit-like serrations, the ectopic crypt foci and the eosinophilic cells with stratified elongated nuclei [51, 52, 61]. TSAs have an unquestionably malignant potential, but the absence of clear cytological atypia, infrequent or absent mitoses, low Ki-67 proliferation index, β-catenin and p53 negativity, and retention of p16 staining, suggest TSAs are not intrinsically dysplastic, but a superimposed dysplasia (conventional and/or serrated) can develops during progression [61]. The molecular features are quite peculiar, indeed TSAs presenting *KRAS* mutation (50–70%) are left-sided, have CIMP-L phenotype and when they acquire high grade dysplasia they also present *TP53* mutations and WNT pathway activation, giving rise to *KRAS*mt/*BRAF*wt/MSS CRCs; while TSAs with *BRAF* mutations (20–40%) are right- or left-sided, have CIMP-L (or intermediate) phenotype and in the subsequent phase acquire *TP53* mutation and WNT activation leading to *KRAS*wt/*BRAF*mt/MSS CRCs [54]. Thus, TSA could be the precursor of the this latter rare and aggressive CRC molecular subtype [61].

Of note, the serrated polyps described above, are not mutually exclusive: they can co-exist in the same patient or even in the same polyp; this could be related to polyps that switch phenotype as they accumulate genetic events, evolving from a kind of lesion to another characterized by greater progression potential or could be the result of different genomic pathways that create a collision lesion [61, 62].

Thanks to all these morphological and molecular data, it is straightforward to understand how *BRAF* mutation is an early event in CRC carcinogenesis. In fact, it is present even in the 63% of “serrated aberrant crypt foci”, the earliest premalignant serrated lesion [63]. Furthermore, testing for ^V600E^*BRAF* in primary CRCs and matched metastases, suggests a good mutational status concordance between primary and secondary lesions and tumors lacking ^V600E^*BRAF* do not acquire this mutation in their metastases [64]. However, *BRAF* mutation alone is not sufficient for malignant transformation [65] as demonstrated by recent studies that point out the pivotal role of WNT signaling hyperactivation in the “serrated pathway” [66] and suggest how mutant *BRAF*, phosphorylating the transcriptional co-repressor MAFG, via the BRAF-MEK-ERK axis, induces CpG-islands hypermethylation [5, 67]; this could be the molecular confirmation of the link between *BRAF* mutation and CIMP-H/MSI status in CRC [68]. These new insights could explain the apparent change of the *BRAF* mutation clinical value during the natural history of CRC.

### *BRAF* mutations and new CRC molecular classifications

Recently, the CRC Subtype Consortium (CRCSC), analyzing and merging different subtyping algorithms based on several gene expression data sets, identified 4 CRC consensus molecular subtypes: CMS1 (14% MSI immune: hypermutated, MSI, with a strong immune activation), CMS2 (37% canonical: epithelial, marked WNT and MYC signaling activation), CMS3 (13% metabolic: epithelial and metabolic dysregulation), and CMS4 (23% mesenchymal: TGF-β activation, stromal invasion and angiogenesis). About 45% of CMS1 group, less than 10% of CMS3/CMS4 and less than 5% of CMS2 groups harbor a *BRAF* mutation, in line with the well-known association between this event and MSI. Of note, our group has recently demonstrated that CMS subgrouping is significantly associated to a prognostic stratification in a large series of Italian *BRAF*mt mtCRCs [69].

However, this classification is not able to explain the high heterogeneous targeted-therapy response in the ^V600E^*BRAF* CRCs subgroup. Hence, according to gene expression profiles, Barras et al. [70] distinguished two subtypes of ^V600E^*BRAF* mutants: BM1 and BM2.

BM1 (one-third of all *BRAF*mt CRCs) is characterized by KRAS/AKT pathway activation, mTOR/4EBP1 deregulation and EMT enhancing [70]. Whereas, in BM2 group (two-thirds of *BRAF*mt CRCs) is present a cell-cycle and cycle checkpoints-related events deregulation [70].

Intriguingly, several molecular differences have been observed between BM1 and BM2 groups. BM1 group displays a stronger immune profile (IL2/STAT5, IL6/JAK/STAT3 pathways activation, TNF-α signaling via NF-kB, and allograft rejection) and an enrichment in angiogenesis and TGF-β-mediated processes; while BM2 group is enriched in metabolic processes and displays high CDK1 and low cyclin-D1 levels [70].

Although BM1 subtype seems to results in a poorer prognosis compared to BM2 subtype, MSI status remain the dominant prognostic factor; in fact, BM classification is independent of MSI status, methylation patterns, *PI3K* mutation, gender and sidedness [70].

Most BM1 and BM2 patients (70%) were classified into CMS1, whereas only a few were found in CMS2 (2%), CMS3 (5%), and CMS4 (17%). Interestingly, all CMS4 *BRAF* mutants are classified as BM1, whereas CMS1 BRAF mutants are distributed into both BM1 and BM2, demonstrating that BM subgroups can refine CMS classification, capturing additional transcriptomics variations within the *BRAF* mutants of the CMS1 group [70].

Even if V600 is the most frequent *BRAF* mutation in CRCs, other non-V600 mutations have been identified by novel and more accurate molecular techniques [36]. Performing functional studies on non-colorectal preclinical models, Yao et al. [71, 72] classified the entire spectrum of *BRAF*-activating mutations according their RAS dependency for signaling and whether they act as a monomer or dimer. Three classes have been identified: class 1 includes the V600 mutations and BRAF protein acts as constitutively active monomer; class 2 consists of kinase active mutations in codons 464, 469, 597 or 601 and BRAF acts as constitutively active dimer; for both class (1 and 2) kinase activity is RAS-independent [71]; class 3 mutations affect codons 287,459,466,467,469,581,594,595 or 596, and BRAF can act as a dimer but has impaired or no kinase activity, so signaling is RAS-depend and remain sensitive to ERK-mediated inhibiting feedback [35, 72].

Non-V600 *BRAF* mutations are present in less than 2% of CRCs and their prognostic and predictive value is not yet well characterized. Conversely to other kinase-activating mutations, class 3 comprises DFG inactivating mutations that increase the heterodimerization of BRAF with wild-type CRAF, inducing an indirect and only modest activation of MAPK pathway, demonstrated by MEK and ERK phosphorylation [45]. Although these mutations confer an impaired kinase activity, they retain an oncogenic potential that could be explained with the co-expression of other molecular alterations; in fact, kinase-dead *BRAF* mutants coexist and synergize with *RAS/EGFR* gain-of-function mutations, while these genomic events are mutually exclusive in other subgroups of CRCs [73].

Hence, CRCs bearing non-V600 *BRAF* mutations constitute a distinct clinico-pathological subset, different from other *BRAF* mutations classes; indeed, class 2 and class 3 CRCs usually are non-mucinous, MSS, arise on the left side of younger male patients, have no peritoneal spread, lower grade at presentation and are related to a more favorable overall survival (OS) rates compared to both ^V600E^*BRAF* mutants and wild-type CRCs [23, 34, 74].

### *BRAF* mutations and MMR status in CRC

Microsatellites are repetitive DNA sequences repeated within genome, in both coding and noncoding regions. MSI is a condition of genetic hyper mutability resulting from defective DNA MMR machinery. It is characterized by clustering of mutations in microsatellites, typically consisting of repeat length alterations. The presence of MSI represents the phenotypic evidence of MMR deregulation [75].

Approximately 15% of CRCs display MSI due to either a germline mutation in MMR genes (genetic MSI, called Hereditary Non-Polyposis Colorectal Cancer or Lynch syndrome, 3%) or a somatic inactivation of one gene of the same group, most commonly through the hypermethylation of *MLH1* promoter region (sporadic MSI, 12%) [76]. MSI prevalence in early stage disease (stage II and III) is about 15% and is related to better OS and disease free survival (DFS) and lower metastatic rate; whereas, there are only limited and inconsistent data regarding MSI prognostic implications in higher stage disease (stage IV) also due to its less prevalence (3–5% of mCRCs) [27, 77]. The predictive role of MSI in early stage is still matter of debate: although with conflicting results, a large amount of preclinical and clinical evidences suggests a possible resistance to fluoropyrimides in these CRC patients predicting a worse response to adjuvant chemotherapy (CT) with fluorouracil-based regimens [78, 79].

The somatic inactivation of MMR genes is strongly associated with ^V600E^*BRAF* mutation (60%), which is virtually absent in Lynch syndrome [80, 81]. Hence, somatic *BRAF* mutation testing has been included into the Lynch syndrome screening algorithm [76, 82–86]. The prognostic and predictive value of coexisting *BRAF* mutation and MSI is still matter of debate.

MSI CRCs share several clinico-pathological features with the *BRAF*mt ones: old age, female sex, right-side, large size, advanced pathologic T stage at diagnosis, mucinous features, poor differentiation, high grade TILs and peritumoral lymphoid reactions (Crohn-like) [15]. Molecular basis of the close relationship between MSI, high grade histology and TILs relies on MMR deficiency that leads to an elevated mutational burden, and wide expression of neoantigens [46]. However, effects of *BRAF* mutational status on immune response remain unclear. In fact, while there are evidences that TILs (especially CD8+ T-cells) are associated with MSI/*BRAF*wt CRCs (Lynch syndrome) [87, 88], this relationship remains unclear in *BRAF*mt CRCs. On the contrary, the presence of a marked peritumoral lymphoid reaction is often present in *BRAF*mt CRCs (regardless to MSI status), but its prognostic value has not yet been fully elucidated; Zlobec et al. [89] suggest that peritumoral-only inflammation and relative lack of TILs might explain part of the poor prognosis of patients with *BRAF*mt CRCs.

It is also possible to recognize some immunohistochemical features related to MMR status and *BRAF* mutations, in particular: a reduction in CDX2 staining, related to adverse prognosis, is present both in *BRAF*mt/MSI and *BRAF*mt/MSS CRCs [90, 91]; CK20 expression is preserved in *BRAF*mt/MSS [30] as in *BRAF*wt, but lost in *BRAF*mt/MSI CRCs CRCs [30, 90, 91]; CK7 is only minimally expressed in CRCs [92] but is frequently upregulated in *BRAF*mt/MSS CRCs [30] interestingly in tumor budding regions [93].

The most common and therefore deeply studied *BRAF*mt CRC subtype is that one catheterized by MSI, but unfrequently *BRAF*mt CRCs retain MSS; this unique subtype is related to poorer outcomes compared to *BRAF*mt/MSI CRCs regardless the stage [25, 28, 82–84], with similar rates of *KRAS*mt/MSS CRCs (both in stage III and in stage IV, even after complete liver metastasectomy) [94, 95]. *BRAF*mt/MSS CRC shares some clinical features with *BRAF*mt/MSI one, like the proximal colon location, but does not have a different gender distribution and usually arises in younger patients. Histologically, like *BRAF*mt/MSI CRC, is mucin-producing and poorly differentiated [31, 96], but presents more aggressive morphological features such as frequent tumor budding, lack of TILs, frequent lymphatic, perineural, and vascular invasion and increased lymph node metastases compared to both *BRAF*mt/MSI and *BRAF*wt CRCs [30, 31]. Molecularly, *BRAF*mt/MSS CRCs have multiple genetic aberrations associated with both “serrated” and “conventional” pathways. Indeed, often displaying *TP53* mutation, correlated with “conventional” pathway and advanced stage but uncommon in MSI CRCs [82–84]; have a comparably high rate of CIN as *BRAF*wt CRCs, though with different patterns (“focal” vs “whole chromosome arms”) [82, 83] indicating the prominent CIN contribution to the progression and poor outcomes [83]. Conversely, *BRAF*mt/MSS CRCs often present hypermethylated genes (at 60%), an infrequent event in “conventional” pathway CRCs (3%) [82, 83], demonstrating that CIMP is prevalent in all *BRAF*mt CRCs, but at a higher frequency in MSI (70–80%) than MSS (60%) cancers [82, 84].

### Prognostic impact of *BRAF* mutations in CRC

Despite new chemotherapeutic regimens and targeted drugs have been approved, patients with *BRAF*mt CRC still have lower response rates to conventional therapies and poorer OS rates regardless of their stage at diagnosis (5-years OS 47.5% vs. 60.7%) [15]. However, is important to consider the clinical and molecular context in which *BRAF* mutations occur and in particular the MMR status at diagnosis.

Indeed, according to several independent studies, patients with ^V600E^*BRAF* CRC have significantly poorer OS compared to patients with *BRAF*wt CRC, regardless MMR status and stage, but only in a univariate analysis and not in multivariate analysis, suggesting the presence of confounding factors [28, 50, 94, 97]. Nevertheless, Samowitz et al. [26] postulated that the negative prognostic value of *BRAF* mutational status is subject to MMR status, regardless of stage, showing that in microsatellite-low (MSI-L)/MSS CRCs *BRAF* mutation was prognostic for poor OS while in MSI-H CRCs *BRAF* mutational status seems to have no significant effect on 5-year OS; similar findings has been reported also by Roth and colleagues [25] for early stage disease (stage II and III). Conversely, other authors reported that *BRAF* mutation with MSI, in early CRC stage, may represent a positive prognostic marker, associated with a lower risk of dissemination [40].

Whereas to date, *BRAF* mutation remains the only oncogenic mutation that predicts poor prognosis in mCRC [15, 50]. Tran et al. [27] reported a strong association between MSI and BRAF mutation and demonstrated poorer OS rates in MSI mCRCs suggesting that, unlike in early stage disease, MSI could represent a negative prognostic factor in advanced disease, although this is driven by its association with *BRAF* mutations.

Even though we are in the in the era of molecular characterization, sometimes *BRAF* testing is not available or reimbursed, but given its great negative prognostic value, in mCRC patients, a simple nomogram to predict ^V600E^*BRAF* mutation in *RAS*wt population has been developed, using gender, primary tumor location (right-sided vs left sided) and histology (mucinous vs non mucinous). mCRC patients with the highest score (right-sided primary, female and mucinous) had a 81% chance to bear ^V600E^*BRAF* mutation [44].

However, the outcomes of patients with *BRAF*mt mCRC, still remain quite heterogeneous [33]. The molecular and clinical basis of these differences could be related to different *BRAF* mutation types, MMR status and to other several genomic events occurring in CRC pathogenesis [34, 70–72]. Indeed, non-V600E *BRAF*mt mCRCs remain a unique subset with better OS compared with both with *BRAF*wt and with ^V600E^*BRAF*mt ones [14, 23].

This complex interaction could also validate some *BRAF*mt mCRCs clinical aspect related to worse prognosis such as the peculiar metastatic spread pattern (peritoneal dissemination and increased number of metastasis) [98].

Therefore, since the remarkable prognostic difference between MSS/*BRAF*mt and MSI/*BRAF*mt CRCs, even in metastatic settings, molecular subtyping according BRAF mutational status alone is an insufficient prognostic index and the assessment of MMR status should always be performed [15]. In addition, the inclusion of other clinical and laboratory criteria could be useful to better prognostically stratify *BRAF* mutant patients as recently demonstrated by Loupakis et al. [99, 100]; items included in this prognostic score are: performance status, CA19.9, LDH, neutrophils/lymphocytes ratio, grading, liver/lung/nodal involvement. Combining these variables authors built both a “complete” prognostic score, and a “simplified” one (excluding laboratory features). Although further validations are needed, this prognostic scoring systems seems to be sufficient to stratify patients in 3 subgroups (low, intermediate and high risk) with significantly different outcomes.

### Predictive impact of *BRAF* mutations in CRC and new therapeutic approaches

If the prognostic value of *BRAF* mutations in CRCs has been widely demonstrated, their predictive value is a question still to be answered.

mCRC patients harboring ^V600E^*BRAF* mutation have worse OS, mostly in the subgroup treated with conventional CT [50]. These data are consistent with several studies where ^V600E^*BRAFmut* patients showed significantly poorer PFS and post-progression survival (P-PS) during first-line CT treatment [101, 102]. Conversely, other studies, even with some limitations, reported that *BRAF* mutational status has a modest or no impact on PFS of first-line CT, but median PFS dramatically declines for *BRAF*mt patients during second and third-line CT treatment [33, 46, 50]. The poor PFS of *BRAF*mt patients could be related to the early development of resistance mechanisms, more rapidly than in *BRAF*wt patients [15].

Mutations in the *RAS* genes (*KRAS* and *NRAS*), the upstream effector of BRAF in RAS-RAF-MAPK and PI3K-AKT-mTOR signalling pathway, are well-recognized biomarkers of resistance to anti-EGFR monoclonal antibodies (MoAbs) [103–107] conversely, the predictive role of *BRAF* mutations in patients receiving targeted-therapies remains uncertain. Several independent studies and metanalysis investigated the predictive role of *BRAF* mutations for anti-EGFR targeted treatments, observing that, like *RAS*, *BRAF* mutations could predict resistance to anti-EGFR therapies [46, 106, 108–110]; on the contrary, a metanalysis including 7 randomized trials evaluating the BRAF mutations effect on anti-EGFR treatment, reported that there are insufficient evidences to definitively confirm that *BRAF*mt patients do not benefit from anti-EGFR therapy [111]. These ambiguous conclusions could depend on the small number of patients enrolled in the studies and on the consequent impossibility to characterize the heterogeneity among the population of *BRAF* mutated patients.

Due to the aggressiveness of *BRAF*mt mCRC, only 60% of patients can receive a second-line CT treatment [20, 33]. Hence, intensive and combined first-line approach with conventional CT and targeted therapies could be a potentially effective strategy, as demonstrated by FOLFOXIRI plus bevacizumab regimen, which demonstrated an improved response rates compared to FOLFIRI in late-stage *BRAF*mt CRC [112], and increased PFS and OS [23, 24, 113, 114]. However, this intensive approach could be limited by increased toxicity in these patients, who typically have a reduced performance status at diagnosis [20, 24, 112, 115, 116].

Several targeted inhibitors against *BRAF* mutations or other key components of MAPK pathway are emerging, but unfortunately, differently from other cancers, *BRAF*mt mCRCs resistance to single targeted-drug regimens is broadly attested in clinical practice. In melanoma, sorafenib, the first tyrosine kinase inhibitor (TKI) of RAF-kinases, achieved only scant clinical effects due to its greater affinity for other kinases besides BRAF [117]; subsequently a TKI able to target ^V600E^*BRAF* mutation was developed, vemurafenib, and in phase III trial demonstrated improved rates of OS and PFS compared to standard CT (dacarbazine) in patients with previously untreated melanoma with the BRAF V600E mutation [118]. In *BRAF*mt mCRC no similar results were achieved using TKI against BRAF [33, 35, 119]. Similar assumptions have been made for dabrafenib, another strong and selective BRAF TKI, whose clinical effect as single agent in metastatic melanoma is limited due to the rapid acquisition of resistance mechanisms [117]. To explain unresponsiveness of mCRC to BRAF inhibition, several resistance mechanisms have been identified: firstly, BRAF inhibition can induce a feedback activation of EGFR, which supports cellular proliferation; interestingly a difference in EGFR expression degree between melanoma and mCRC cells has been observed, which could partially explain the difference in clinical response to BRAF inhibition [119]. Moreover, BRAF inhibition stimulates an ERK-dependent activating feedback on EGFR, with a MAPK pathway reactivation through CRAF and the hetero-dimerization between CRAF and BRAF, making monomeric BRAF-inhibitors ineffective [71, 120]. Other mechanisms of acquired resistance include: ERK-mediated gain-of-function, *MEK1* mutations, *BRAF* amplification and *KRAS* alterations [19, 121]. Finally, PI3K pathway crosstalk-activation through KRAS, as well as mutations in *PIK3CA* and *PTEN*, confers cancer cells resistance to MAPK inhibition [122].

Hence, it is necessary to introduce a broader combinatorial therapy, using targeted drugs acting on multiple critical biological processes for the neoplastic cells, like the combination of BRAF-/MEK-/ERK-inhibitors, with anti-EGFR MoAbs and/or standard CT agents. Considering the rationale for this strategy, several exploratory studies investigating combination therapies have been conducted, showing variable but overall favorable clinical response rates in *BRAF*mt mCRCs [123–125]. Furthermore, the phase III trial BEACON has recently proved a significant survival advantage for the combination of encorafenib plus cetuximab or the same doublet plus binimetinib compared to current standard treatments [126, 127]. This seminal study will pave the way for innovative BRAF-specific therapeutic options.

One of the less explored aspect is the potential predictive value of the different *BRAF* mutation types. Actually, in vitro assays validated a BM1 cell lines sensitivity to BRAF-, MEK- and BCL2-inhibitor and a BM2 cell lines sensitivity to CDK1 inhibition [70]. In addition, functional *in vitro* studies conducted on melanoma models showed that FDA-approved BRAF inhibitors vemurafenib and dabrafenib are only effective against class 1 monomer-type mutations (V600 mutations); whereas, class 2 is sensitive to novel panRAF-inhibitor (LY3009120) or MEK-inhibitors (trametininb) and class 3 to EGFR-inhibitor (cetuximab and panitumumab) and RTKs-inhibitors (dasatinib) [34, 71, 128].

Consistent with in-vitro data, BM subtypes and *BRAF* mutations classes might differ in drug-response even in clinical settings, validating the heterogeneous targeted-therapies response in *BRAF*mt patients cohort and supporting prospective testing of novel drug combinations in selected patients subsets [70].

## Conclusion and future directions

Although *BRAF* mutation is a relatively rare finding in mCRC, it is characterized by a critical negative impact in clinical presentation, histology, molecular features, patient outcomes and therapeutic strategies. This is, however, strictly related and dependent on the genetic and epigenetic background that could evolve during disease and therapy, differently from other *BRAF* mutated malignancies. In fact, *BRAF* mutation frequency in early staged CRC is probably underestimated and its prognostic value in this setting remains unclear as demonstrated by poor and contradictory data published to date.

The predictive value of *BRAF* mutation in mCRCs is still blurred; patients bearing this alteration are likely to not respond to therapeutic schemes based on a single TKI; on the contrary, different combinatorial therapies could have an heterogeneous response pinpointing an inter and intra-tumoral heterogeneity. Although several mechanisms of TKI resistance have been investigated, we are still far from a deep molecular comprehension of *BRAF*mt CRCs.

More efforts are needed to provide the knowledge for a rational use of targeted and combined therapies, in order to minimize the selective pressure of resistant clones and reduce side effects. So, it could be useful to further classify and stratify the *BRAF* mutant population, in order to improve the efficacy of personalized therapies.

## Data Availability

Not applicable
